# Effect of distance measures on confidences of t-SNE embeddings and its implications on clustering for scRNA-seq data

**DOI:** 10.1038/s41598-023-32966-x

**Published:** 2023-04-21

**Authors:** Busra Ozgode Yigin, Gorkem Saygili

**Affiliations:** Cognitive Sciences and Artificial Intelligence, Tilburg School of Humanities and Digital Sciences, Tilburg University, Warandelaan 2, 5037 AB Tilburg, The Netherlands

**Keywords:** Computational biology and bioinformatics, Computational models, Data processing, Machine learning, RNA sequencing

## Abstract

Arguably one of the most famous dimensionality reduction algorithms of today is t-distributed stochastic neighbor embedding (t-SNE). Although being widely used for the visualization of scRNA-seq data, it is prone to errors as any algorithm and may lead to inaccurate interpretations of the visualized data. A reasonable way to avoid misinterpretations is to quantify the reliability of the visualizations. The focus of this work is first to find the best possible way to predict sample-based confidence scores for t-SNE embeddings and next, to use these confidence scores to improve the clustering algorithms. We adopt an RF regression algorithm using seven distance measures as features for having the sample-based confidence scores with a variety of different distance measures. The best configuration is used to assess the clustering improvement using K-means and Density-Based Spatial Clustering of Applications with Noise (DBSCAN) based on Adjusted Rank Index (ARI), Normalized Mutual Information (NMI), and accuracy (ACC) scores. The experimental results show that distance measures have a considerable effect on the precision of confidence scores and clustering performance can be improved substantially if these confidence scores are incorporated before the clustering algorithm. Our findings reveal the usefulness of these confidence scores on downstream analyses for scRNA-seq data.

## Introduction

It is now possible, thanks to the advances in sequencing technology for genetic materials, to explore the gene expression profile of individual cells at a higher coverage and greater resolution of the dynamic nature of the transcriptome. Single-cell RNA sequencing (scRNA-seq) provides unprecedented opportunities for extracting underlying biological information from large amounts of data. Consequently, it caused an exponential increase in the number of cells analyzed and triggered the need for efficient computational methods. Effective computational analysis approaches have been developed in recent years, involving several steps such as quality control, mapping, quantification, dimensionality reduction, clustering, finding trajectories, and identifying differentially expressed genes^1^.

On the other hand, scRNA-seq technology suffers from crucial technical challenges, such as high dimensionality and sparse representation of the true transcriptome of single cells^2^. The high dimensionality of scRNA-seq data prevents visual exploration and hampers downstream analyses such as clustering and lineage inference. Dimensionality reduction (DR) methods have been employed to alleviate this problem by creating faithful, lower dimensional representations of the data, so-called embeddings. Commonly used DR algorithms in scRNA-seq include principal component analysis (PCA), t-distributed stochastic neighbor embedding (t-SNE), uniform manifold approximation and projection (UMAP), as well as other variations of these three^3^. Among these DR algorithms, t-SNE stood up as one of the top performers in terms of accuracy and computing cost^4^.

Despite its crucial role in scRNA-seq analysis and impressive performance, t-SNE might not always produce precisely accurate representations, containing some erroneously embedded samples that could lead to inaccurate interpretations of the research’s findings. Generally, ranking-based metrics or human judgment, which may be biased depending on the level of expertise, are used to evaluate the quality of the embeddings. Ranking-based metrics^5,6^ focus on retaining local neighborhood rankings in high and low dimensions instead of considering the preservation of ground truth target labels (label-based). There are also some label-based error detection and confidence estimation methods that have been developed specifically for t-SNE embeddings^7,8^, in a similar way to those in many other domains such as medical image registration^9,10^ and stereo matching^11^. What makes the label-based confidence estimation algorithm^8^ unique is that it generates confidence scores for each and every sample in a t-SNE embedding with a supervised Random Forest (RF) regression algorithm based on target class labels. The six different distance measures utilized as features for the regressor were not chosen domain-specifically, but rather common ones. We argue based on previous studies^12–16^ that the choice of distance measures can have a strong influence on the overall performance of the algorithm. As our first contribution, we explored the contribution of 28 different distance measures to predict the sample-based confidence scores for t-SNE embeddings and found the best possible distance measures to be used in estimating confidence scores from the embedding, particularly on scRNA-seq data.

t-SNE is one of the key elements in the downstream analysis of scRNA-seq data, such as clustering. Although providing impressive results in terms of detecting unreliable samples in the embeddings, these scores have not been shown to be effective for clustering. As our second contribution, we showed that these confidence scores can be used to further increase the performance of K-means^17^ and Density-Based Spatial Clustering of Applications with Noise (DBSCAN)^18^ clustering algorithms.

## Results

### Seven top performing distance measures revealed based on performance

Our first task is to find out the performance of 28 different distance measures to predict the sample-based confidence scores for t-SNE embeddings. The distance measures are presented in Table 1. In accordance with^8^, we trained an RF regressor on the training sets of the AMB18 and Baron Human datasets by using each distance measure individually as a feature. Next, we evaluate the performance of each distance measure with intra-dataset experiments. We predicted the confidence scores of each sample in the embedding and sorted them in descending order. We calculated the score indicating the number of errors in the last 100, 50, and 10 samples with the lowest confidence scores. Both Neighbor Preservation Ratio (NPR)^19^ and RF scores for Baron Human and AMB18 datasets are shown in Table 1. After considering the individual performances of distance measures on both datasets and also their proven success in high-dimensional spaces, the seven best distance measures, Braycurtis, Correlation, Cosine, Kullback-Leibler, Pearson, and WIAD, were chosen.

**Table 1:** The number of erroneous samples that can be correctly detected in the 100, 50, and 10 lowest NPR scores, and RF scores obtained by training on Baron Human and AMB18 and testing on their test sets with individual distance measures. Distance measures that perform well jointly in both datasets are marked in bold.

Distance Measures	For Prediction on Baron Human			For Prediction on AMB18		
	100	50	10	100	50	10
**Braycurtis**	31	21	7	10	6	4
**Correlation**	25	19	8	11	8	4
**Cosine**	26	20	8	9	7	4
**Dice**	32	20	6	12	9	4
**Kullback-leibler**	28	20	5	13	9	6
**Pearson**	25	19	8	11	8	4
**WIAD**	29	23	7	12	9	4
Average distance	11	9	3	11	9	5
Average euclidean	6	4	2	11	6	4
Canberra	12	6	2	7	6	4
Chebyshev	15	9	4	7	6	4
Chi-squared	16	11	4	12	10	6
Chord	22	17	8	8	7	3
Clark	7	5	1	6	6	4
Divergence	5	1	1	8	5	4
Euclidean	6	4	2	11	6	4
Hassanat	9	5	3	7	6	3
Jaccard	27	17	6	10	9	4
Jeffreys	19	15	4	11	10	6
Jensen difference	15	12	3	10	10	6
Lorentzian	15	8	4	12	8	6
Manhattan	12	9	4	12	9	4
Mean censored euclidean	6	4	2	11	8	3
Motyka	29	21	7	9	6	4
Squared euclidean	8	6	2	12	10	5
Squared chord	14	12	3	11	9	4
Squared pearson	23	18	9	8	6	4
Vicis symmetric	15	8	1	5	4	2

### RF with chosen distance measures detected erroneous samples in the embedding better than the previous model and NPR

We trained an RF regressor by using grid search for fine-tuning on the two different training sets, AMB18 and Baron Human, together with their generated confidence scores, and used this algorithm to predict the confidence scores of the other datasets (inter- and intra-datasets). In this study, we examined how well our suggested model performed against the model created on two distinct domains and different distance measures by Yigin et al.^8^. We kept the same split for train and test sets (ratio of 80/20) in the shared datasets for both experiments to have a fair comparison, and we kept the same performance evaluation method.

To systematically assess the performance of the confidence estimation algorithm, the number of successfully detected erroneous samples in the lowest 100, 50, and 10 NPR-scored and RF-scored samples were calculated and summarized in Table 2. Since the numbers show successfully-revealed erroneous samples in the embedding, the higher they are, the better the performance. In this table, the total number of erroneous samples for each dataset was also provided since the ratio of the correctly detected erroneous samples could be useful for interpreting the performance differences between the datasets. In most cases, our predictions generally showed higher concordance to the ground truth erroneous samples in comparison to Yigin et al.^8^ and the NPR score. It is noteworthy that the success of detecting erroneous samples has increased at a much higher rate than^8^ particularly in the lowest 10 RF-scored samples, even occasionally a success of 10 out of 10 had been attained. These results signify the importance of the chosen distance measures in predicting confidence measures accurately.

**Figure 1:**
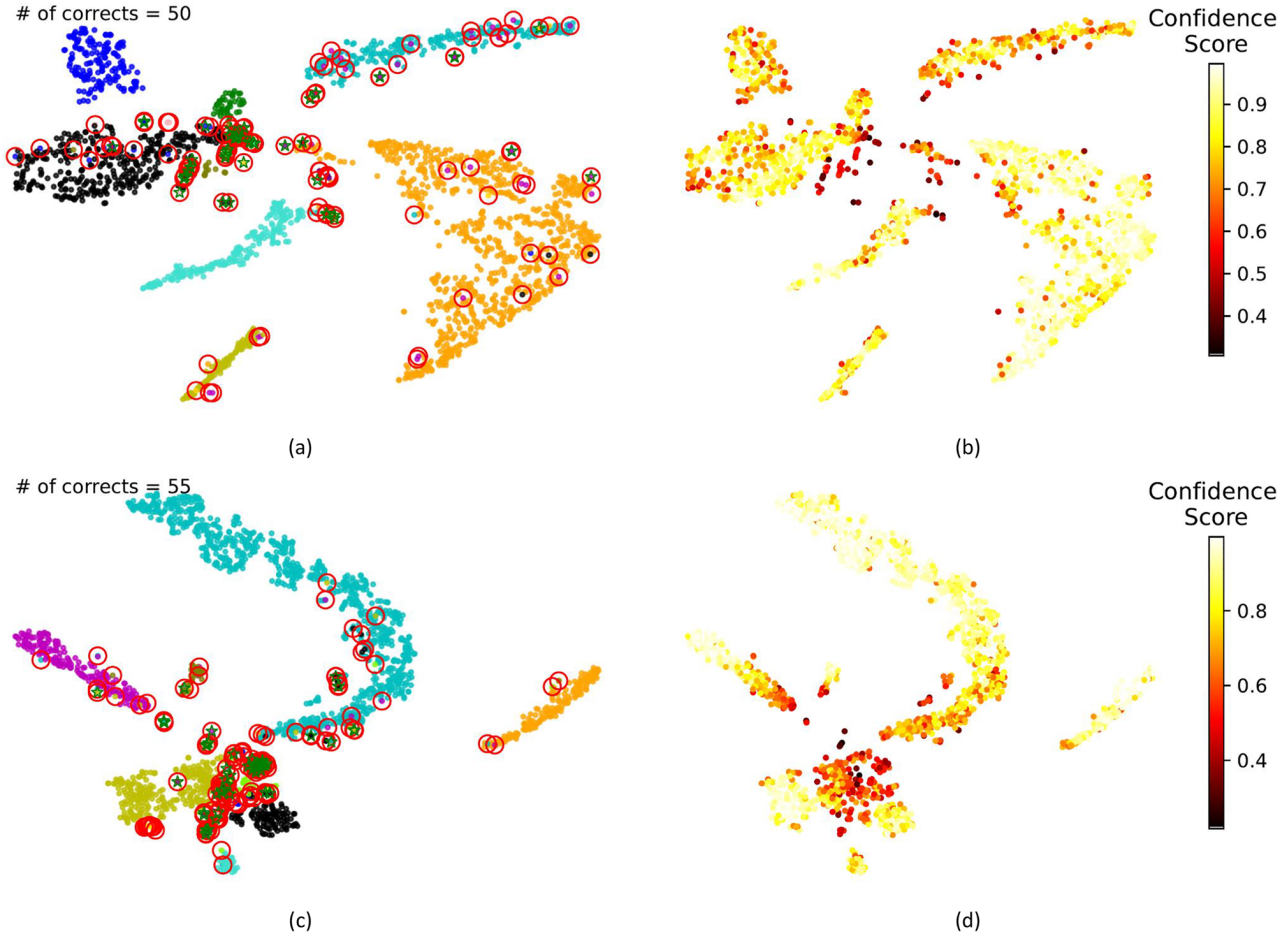
t-SNE embeddings of the Segerstolpe (top) and Baron Mouse (bottom) datasets (**a**) and (**c**) Erroneous samples are marked with red circles, correctly detected erroneous samples are marked with green stars (**b**) and (**d**) Heat maps show the confidence scores of each sample in the embeddings.

In addition to the quantitative results, Fig. 1 shows t-SNE visualizations of the Segertsolpe and Baron Mouse datasets, respectively. Cells were color-coded based on their cell-type annotation in Fig. 1a,c. While red circles represent the erroneously embedded samples, green stars reflect the correctly detected erroneous samples by the confidence estimation algorithm. Predicted erroneous samples of the confidence estimation algorithm are calculated over 100 samples with the lowest confidence score, and the predicted erroneous samples that match the ground truth erroneous samples are marked with stars. Figure 1b,d show heat maps of the distribution of confidence scores over the same t-SNE distributions. It is also perceptually apparent that the erroneous samples indicated in Fig. 1a,c have relatively lower confidence scores than other samples.

### Clustering performance with K-Means and DBSCAN algorithms improved after the elimination of the low-confident samples

**Figure 2:**
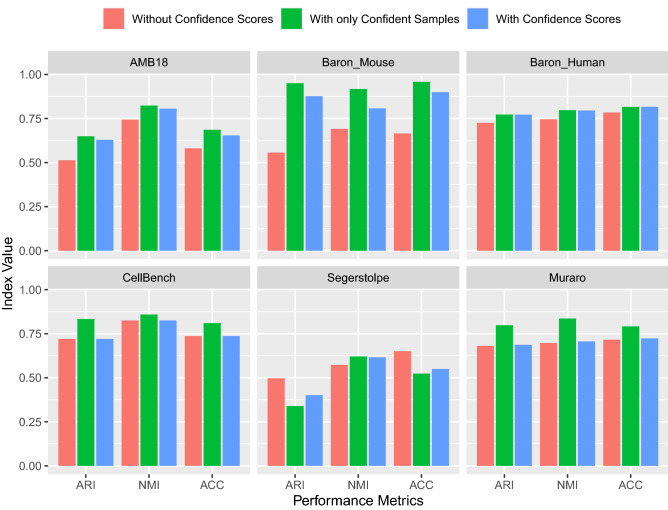
Evaluation of the K-Means clustering performance on the whole dataset without confidence scores, on the dataset with only high-confident samples, and on the dataset where the eliminated samples are then positioned on the existing clusters, respectively. The elimination rate is chosen as 0.4. This elimination rate corresponds to the threshold values of confidence scores of 0.985, 0.824, 0.946, 0.859, 0.825, and 0.873 for the datasets, respectively.

**Figure 3:**
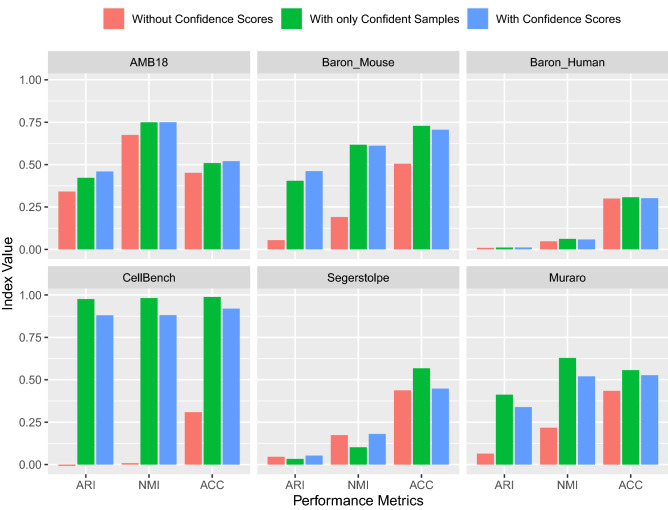
Evaluation of the DBSCAN clustering performance on the whole dataset without confidence scores, on the dataset with only high-confident samples, and on the dataset where the eliminated samples are then positioned on the existing clusters, respectively. The elimination rate is chosen as 0.4. This elimination rate corresponds to the threshold values of confidence scores of 0.985, 0.824, 0.946, 0.859, 0.825, and 0.873 for the datasets, respectively.

Although providing exciting results, the confidence measures for the embeddings have not yet been used in the downstream analysis. Their contribution is yet to be shown in particular for clustering. To investigate if using confidence scores can improve the performance of downstream analysis for scRNA-seq data, we employed two clustering methods, K-Means^17^, and DBSCAN^18^, before and after eliminating the low-confident samples corresponding to 40%(0.4), 30%, 20%, and 10% of the data (as explained in Section "Investigation of the impact of confidence scores on clustering algorithms"). We subsequently applied these four versions of implementation to our collection of scRNA-seq datasets and compared their performance using evaluation measures, including ARI, NMI, and ACC. The results of the K-Means and DBSCAN clustering algorithms with the elimination rate of 0.4 are shown in Figs. 2 and 3, respectively. The evaluation results for each elimination rate are summarized in Fig. S1 and Fig. S2 for both clustering algorithms in the supplementary material. The results with various elimination rates show that the best performance improvement was obtained with the elimination rate of 0.4. While it may appear prudent to select an elimination rate of 0.4 for this study, we leave the choose of elimination rate to the user’s choice as a hyperparameter that should be set in accordance with the sensitivity of the targeted study. In most cases for both clustering algorithms, the inclusion of confidence scores substantially increased the clustering performance.

## Discussion

The ultra-high throughput of scRNA-seq techniques has resulted in various new computational challenges, such as normalization, dimensionality reduction, clustering, and differential expression analysis. Given the large impact of the assessment of dimensionality reduction on downstream analysis for meaningful biological discoveries, it is important to find a quantification method that is applicable to any kind of embedding. In this work, we presented a RF-based confidence estimation algorithm for predicting the confidence scores of each sample in the embeddings. We demonstrated that confidence scores could be utilized to enhance the performance of the clustering of scRNA-seq data.

Using a variety of distance measures, in line with the studies in the literature, we demonstrated that the choice of a similarity measure has a significant impact on the confidence estimation. In our comparison of distance measures specifically to scRNA-seq data, correlation-based measures (Correlation, Pearson, etc.) outperformed distance-based measures (Euclidean, Manhattan, Chebyshev, etc.), which is in line with the results of^16^.

The experimental results in Table 2 show that the proposed method detects erroneous samples in the embedding better than the previous model and NPR-scored results. The main reason why the results from the previous study performed worse than the proposed algorithm is that the choice of the distance measures for confidence estimation was not made domain-specifically. The resulting improvement clearly demonstrates the significance of appropriate distance measure selection. Although the proposed algorithm performs better in all datasets compared to the NPR score, it is seen that the proposed algorithm has a slightly lower number of correctly detected erroneous samples for the AMB18, CellBench, and Muraro datasets, which inherently have a lower number of erroneous samples in their embeddings.

**Table 2:** The number of erroneous samples in the 100, 50, and 10 lowest confidence scores based on our top seven distance measures, scores from^8^ and the NPR scores. RF scores are obtained from training on AMB18 and Baron Human datasets and tested on all test sets. Bold numbers indicate the best results for each test set. The top performances are indicated in bold.

	Test set	Total number of Erroneous samples	Results of proposed approach			Results of ^8^			Results of NPR		
			100	50	10	100	50	10	100	50	10
Train on AMB18	AMB18	31	7	6	4	**13**	**8**	0	4	3	2
	Baron Mouse	144	**45**	**33**	**9**	19	12	2	33	16	3
	Baron Human	91	**44**	**30**	**9**	21	15	4	21	9	4
	CellBench	105	**42**	**28**	**8**	36	17	4	35	14	3
	Segerstolpe	116	**47**	**30**	**10**	37	25	6	29	19	3
	Muraro	60	**32**	**25**	**8**	27	15	4	17	13	7
Train on Baron Human	AMB18	31	8	4	**3**	**9**	**6**	1	4	3	2
	Baron Mouse	144	**55**	35	**10**	47	32	9	33	16	3
	Baron Human	91	**39**	**29**	**10**	36	19	7	21	9	4
	CellBench	105	25	18	4	**36**	**26**	**8**	35	14	3
	Segerstolpe	116	**50**	**34**	**10**	37	25	7	29	19	6
	Muraro	60	16	8	2	**22**	**17**	**8**	17	13	7

As seen in Fig. 1, the proposed confidence estimation algorithm tends to find erroneous samples that are in inter-cluster transition regions rather than cluster centers. On the other hand, for the datasets with a small number of erroneous samples which causes the problem of imbalanced data, the confidence estimation algorithm tends to produce higher confidence scores. This situation prevents the selection of a gold standard elimination rate for the elimination of the low-confident samples.

The performance improvement obtained when the confidence scores are integrated into the DBSCAN algorithm is higher than that of the K-Means method, as can be seen in Figs. 2 and 3. We observed that when we eliminate the low-confident samples, it facilitates the determination of the optimal values of the parameters of the DBSCAN algorithm, therefore it may help to produce better clusters with high NMI, ARI, and ACC values. We argue that the better performance increase in DBSCAN compared to K-means is due to the difference in the number of parameters that need to be adjusted. Two hyperparameters of the DBSCAN algorithm, $\epsilon$, and MinPts can be set more effectively with confident samples than the only parameter, *K*, of the K-Means algorithm. Furthermore, the performance increase difference among the datasets can be related to the sample distribution of the datasets. For instance, the Segerstolpe dataset has a more imbalanced class distribution than the CellBench dataset, which results in the elimination of minority class samples. As a consequence, the clustering performance improvement is significantly lower.

## Methods

The t-SNE algorithm proposed by Maaten et al.^20^ is used to obtain lower-dimensional representations from high-dimensional datasets. We utilized the t-SNE implementation of Scikit-learn with default values of 30 for perplexity and 2 for the number of components.

### Definition of the erroneous/correct samples and confidence scores according to the local neighborhood

Using the same approach of Yigin et al.^8^, by looking at the local neighborhood in an embedding, we checked whether each sample in embedding shared the same label with the majority of the samples in its nearest neighborhood ($K=20$ selected). A sample is considered correctly embedded if it has the same label as at least *K*/2 (10 in our case) neighbors, and erroneously embedded if it has a different label than the majority.

Similarly, we obtain ground truth confidence scores by calculating the ratio of its neighbors which has the same label as the sample itself. The confidence values thus are generated in a range between 0 and 1, with 0 denoting the lowest possible confidence and 1 representing the highest possible confidence.

### Neighborhood preservation ratio (NPR)

NPR is a metric for comparing the performance of the dimensionality reduction outcomes and was also utilized by Maaten et al.^19^. NPR quantifies the extent to which nearest-neighbor distances in the original space are correctly preserved in low-dimensional space based on Euclidean distances. We used the NPR scores as a baseline to compare with our confidence estimations. To calculate NPR, similar to in^8^, we calculated the intersection amount of the closest *K* neighbors in the original and low-dimensional space. For each point *i*, we selected the *K* lowest Euclidean distances in both the low-dimensional space ($N_d(x_i,1:K+1)$) and the original space ($N_D(x_i,1:K+1)$). The NPR is the ratio of the number of the preserved neighborhood:

1 $$\begin{aligned} NPR (x_i) = \frac{N_D(x_i,1:K+1)\cap N_d(x_i,1:K+1)}{K} \end{aligned}$$

where *K* is the chosen number of nearest neighbors and $N_D(x_i,1:K+1)\cap N_d(x_i,1:K+1)$ calculates the number of co-existing points in original and low-dimensional spaces.

### Extraction of the distance-based features for the investigation of their impact on confidence estimation

For the estimation of the confidence, the distances between the neighbors in original and low-dimensional spaces can be identified by different distance measures. Several studies have been conducted to analyze the performance of the algorithms that are affected by the choice of distance measures, such as the k-nearest neighbor (KNN) classifier^12,13^, image recognition^14^, and some clustering algorithms^15,16^. All these studies conclude that the choice of distance measures has a substantial impact on the performance of these algorithms since they found considerable variations in the results for different distances. They also confirm that no single distance measure can be optimized for all datasets and that the appropriate distance measures for a given study should be determined specifically for the domain containing as much similar data as possible^13^. In^8^, the most common distance measures namely Euclidean, cosine, correlation, Chebyshev, Canberra, and Braycurtis were used to extract features from the datasets from different domains for the prediction of confidence scores. However, there has been yet to examine the effect of different measures on the performance of the confidence estimation algorithm. In this study, we attempted to bridge this gap by examining a wide range of distance measures particularly on scRNA-seq datasets, in order to investigate the distance measures that yield the best confidence estimation results.

In the review presented by Abu Alfeilat et al.^12^, the performance of KNN classifiers using 54 different distance measures was analyzed on 28 different datasets.These distance measures were classified as part of the eight major distance families. Distance measures that we used in this study were chosen among the distance measures used in this review according to their performances. While an accuracy value over 0.75 was typically used as a selection criterion, some exceptional decisions were made, such as excluding measures that were very similar or including the highest performers for each category. In addition, the mutual information similarity measure, which was not included in the review, was also included in this study for comparison because of its high performance in various other tasks. While comprehensive information on distance measures can be found in^12^, Table S1 in supplementary material contains a list of all distance measurements employed in this work along with an explanation for their selection.

We initially evaluated the individual performances of each of the 28 distance measures on the AMB18 and Baron Human datasets in order to determine the best-performing distance measures in these datasets. Using only one distance measure at a time as a feature of the model, we trained and evaluated the confidence estimation algorithm. We extracted the features from a neighborhood of K around each sample by choosing *K* as 20. We used the same approach as Yigin et al.^8^ to calculate distances between the neighbors in original and low-dimensional spaces. First, we sorted the nearest neighbors in both spaces according to the Euclidean distances, and then all 28 distances between them were calculated separately.

The selection of distance measures for the algorithm was based on not only their performance but also their proven success in high-dimensional spaces as demonstrated by numerous studies in the literature. Since the calculation of distance measures constituted the most computationally expensive part of the algorithm, we aimed to limit the number of used distance measures. Many studies have demonstrated the functionality of Cosine similarity and Kullback-Leibler divergence in clustering, as they can effectively measure the (dis)similarity of clusters, especially when dealing with high-dimensional data, such as natural language processing applications and sc-RNA seq analysis^21–23^. Therefore, despite the availability of other distance measures that demonstrate comparable performance, such as Jaccard and Motyka, these two measures have been specifically included in the study. After selecting the joint best performer distance measures on both AMB18 and Baron Human datasets, we concatenated seven distance measures in order to feed them as all input features of the algorithm. These joint best distance measures are Braycurtis, Correlation, Cosine, Dice, Kullback-Leibler, Pearson, and Whittaker’s index of association distance (WIAD).

### RF-based confidence estimation algorithm

We trained an RF regressor to predict the confidence scores by using distance measures as our features and ground-truth confidence scores as our targets. A decision tree framework and ensemble learning techniques were combined in the RF regressor to produce many randomly selected decision trees from the data, which were then averaged to produce a new result that frequently yields accurate predictions, thus preventing overfitting. We utilized the sklearn module to train the RF regression model on the training sets of the AMB18 and Baron Human datasets separately and evaluated our model on the test set of the same dataset (intra-dataset) and on the test sets of other datasets (inter-dataset). We kept the model structure the same, performed 3-fold cross-validation, and conducted grid search for hyperparameter tuning by adhering to the same hyperparameter grid in order to obtain results that are comparable to those of Yigin et al.^8^.

### Investigation of the impact of confidence scores on clustering algorithms

We performed clustering experiments on all datasets that we used in the confidence estimation task. We first reduced the dimensionality of the data to 30 with PCA and then apply the clustering algorithms. In these experiments, we ranked the estimated confidence values between 0 to 1 to obtain four different versions of all datasets eliminating the least reliable samples of $40\%$, $30\%$, $20\%$, and $10\%$. We observed the differences in the performance of the K-Means and Density-Based Spatial Clustering of Applications with Noise (DBSCAN) clustering algorithms, which are frequently used clustering algorithms for scRNA-seq analysis.

We measured the clustering quality of the original dataset, in which potential deviations may occur. Then we obtained confidence values for each sample and removed the low-confident samples according to a certain elimination rate and measured the clustering performance of this data. (With only Confident Samples label in Figs. 2 and  3). After the cluster centers were determined with only high-confident samples, the eliminated samples were again assigned to the clusters according to their distances from cluster centers (With Confidence Scores label in Figs. 2 and 3). In this way, we were able to compare the quality of clusters with the entire dataset without using confidence scores, with the dataset that consists of only high-confident samples after the elimination of low-confident samples, and with the data after positioning the eliminated low-confident samples on the existing clusters.

Clustering results can vary considerably depending on the values of their hyperparameters. We employed a couple of criteria to provide clustering consistency among our comparisons. The most essential hyperparameter of the K-means algorithm is the number of clusters, called K. To determine the optimal K value, we applied the elbow method widely adopted in clustering analysis. It depicts the sum of squared errors as a function of K to search the elbow point. We employ the KneeLocator function from the Kneed package, which was proposed by Satopaa et al.^24^, to find the location with the optimal cluster number K.

The essential hyperparameters for the DBSCAN algorithm are epsilon ($\epsilon$) and the minimum number of points (MinPts) values. To determine the optimal $\epsilon$ value for DBSCAN, we use the method proposed by Rahmah et al.^25^. In this method, first, the average distance between each point and its *k* nearest neighbors is calculated, and then it is plotted in ascending order. The optimal $\epsilon$ value is determined by finding the point of maximum curvature in the ascending curve in the graph with the KneeLocator function. On the other hand, there is no way to determine the value of MinPts automatically as the $\epsilon$ value. There are only a few general guidelines for choosing the MinPts value: (1) Sander et al.^26^ suggested selecting $MinPts = 2*d$, where *d* is the dimension of the dataset, and (2) it is suggested that the MinPts value should be increased with the size of the dataset. Therefore we simply selected $MinPts = d*2 + round(ns/1000)$, where *d* is the dimension of the dataset and *ns* is the number of samples of the dataset. We automatically set these hyperparameters for both clustering algorithms, to observe the clustering performance before and after the elimination of the low-confident samples.

We used three indexes for cluster quality assessment: the Adjusted Rank Index (ARI), Normalized Mutual Information (NMI), and clustering accuracy (ACC). ARI^27^ and NMI^28^ are both commonly used indices for the measurement of partitions’ diversity and quality. We calculated ARI and NMI scores using sklearn.metrics.adjusted_rand_score, and sklearn.metrics.normalized_mutual_info_score from the Scikit-learn library, respectively. It is necessary to modify the accuracy formula used in the classification method for clustering since the clustering algorithm does not provide a relationship between the predicted cluster labels and the ground truth class labels. To calculate the accuracy of clustering results, a confusion matrix with random order is generated. Then accuracy can be calculated by reordering the rows (or columns) of the confusion matrix using the Hungarian algorithm^29^ so that the sum of the diagonal values is maximal. We use the accuracy function from coclust.evaluation module^30^ that adopts the same approach to calculate the accuracy value.

### Datasets

In this study, we analyzed five scRNA-seq datasets, which include mouse brain cells and human and mouse pancreatic cells. All datasets used in this study are provided by Abdelaal et al.^31^, which is available through the Zenodo repository (https://doi.org/10.5281/zenodo.3357167) except AMB18 which is provided by Michielsen et al.^32^. A brief description of each dataset is shown in Table 3. During the experiments, we used the AMB18 and Baron Human datasets both for training and testing by splitting $20\%$ of the dataset for testing and the rest for training. All other datasets were only used for testing.

Datasets had been preprocessed by Abdelaal et al. as explained in^31^. Following their methodology, a CPM (Counts Per Million) read count normalization and $log2(count+1)$ transformation were applied to the data before clustering experiments.

**Table 3:** scRNA-seq datasets used in this study.

Dataset Name	Description of dataset	Number of genes	Number of cells	Number of class
Allen Mouse Brain (AMB18)^32,33^	SMART-Seq V4 samples from the primary visual cortex of the mouse brain	2000	12,771	15
Baron Mouse^31,34^	inDrop samples from mouse pancreatic cells	14,861	1886	13
Baron Human^31,34^	inDrop samples from human pancreatic cells	17,499	8569	14
CellBench^31,35^	CEL-Seq2 samples from lung cancer cell lines, concatenated from three different datasets	12,627	570	5
Segerstolpe^31,36^	SMART-Seq2 samples from human pancreatic cells	22,757	2133	13
Muraro^31,37^	CEL-seq2 samples from human pancreatic cells	18,915	2122	9

## Conclusion

The advancement of the sequencing technologies enables to produce ever growing data sets containing RNA expression levels for thousands of genes and up to millions of cells. A common approach in downstream analysis pipelines for scRNA-seq data is dimensionality reduction, which is typically performed using t-SNE for visualising the data in two dimensions. Although it generally works well in revealing local structure in high-dimensional data, it is prone to errors as any algorithm is and may result in potentially misleading interpretations. Here, we develop a model that assigns confidence scores to each sample in the embedding in order to prevent these misleading interpretations as well as to make the subsequent analysis steps more reliable.

In this study, we showed that domain-specific selection of the appropriate distance measures for the confidence estimation algorithm can substantially improve the success of capturing erroneously embedded samples from t-SNE embeddings. Furthermore, we examined clustering algorithms, one of the downstream analysis steps, as a possible application of confidence scores and showed that confidence score information may improve clustering performance, as well.

This study has contributed to the current research by filling a gap for the use of confidence scores, specifically that of downstream analysis of scRNA-seq analysis. While we concentrated on single-cell transcriptomics data in this study, the confidence estimation algorithm is more broadly applicable to any dataset from other domains after looking for the most suitable domain-specific distance measures. Although we concentrated on t-SNE embeddings in this study, the approach we developed has the potential to be successful with different dimensionality reduction algorithms. The proposed approach can be used to develop a novel adaptive clustering algorithm that makes use of these confidence scores as a feedback to generate clusters. Furthermore, proposed approach can be further optimized by exploring more advanced machine learning methods. Overall, we believe that our approach provides a valuable contribution to the field of scRNA-seq data analysis and has potential for broader applications in other domains.

## Supplementary Information

Supplementary Information 1.

## Data Availability

All datasets analyzed in the current study are publicly available and can be downloaded from Zenodo repository^31^. They can be also downloaded from their public accessions, including AMB18^33^ GSE115746, Baron Mouse^34^ GSE84133, Baron Human^34^ GSE84133, CellBench CEL-Seq2^35^ (GSM3618022, GSM3618023, GSM3618024), Segerstolpe^36^ E-MTAB-5061, Muraro^37^ GSE85241.

## Notes

The codes used in this study are available in the Github repository, at https://github.com/gsaygili/CET.git.
