# Supplementary material for: Effect of distance measures on confidences of t-SNE embeddings and its implications on clustering for scRNA-seq data
Source: Sci Rep. 2023 Apr 21;13:6567. doi: 10.1038/s41598-023-32966-x (PMC10121641; doi:10.1038/s41598-023-32966-x)
Supplement: Supplementary file 1 — Supplementary Information 1. [file 41598_2023_32966_MOESM1_ESM.pdf]

Effect of Distance Measures on Confidences of t-SNE Embeddings and Its Implications on Clustering for scRNA-seq data: Supplementary Information

BUSRA OZGODEYIGIN[1,*] AND GORKEM SAYGILI[1]

[1] *Cognitive Sciences and Artificial Intelligence, Tilburg School of Humanities and Digital Sciences, Tilburg University, Warandelaan 2, Tilburg, 5037 AB, The Netherlands*
[*] *b.ozgodeyigin@tilburguniversity.edu*

1. SELECTION OF DISTANCE MEASURES

Table S1: List of observed distance measures for the selection of reliable distance measures together with the explanation of chosen and discarded. Those marked in bold are included in this study.

Distance Measure	Explanation
**Minkovski DMs**	
**Euclidean**	Accuracy over 0.75
**Manhattan**	The best performance in this group
**Chebyshev**	Accuracy over 0.75
L_1 **DMs**	
**Lorentzian**	Accuracy over 0.75
**Canberra**	Accuracy over 0.75
**Braycurtis**	Accuracy over 0.75
Soergel	Very similar to Jaccard Distance
Kulczynski	Low performance
Mean Character	Very similar to Manhattan Distance
Non Intersection	Very similar to Manhattan Distance
**Inner Product DMs**	
**Cosine**	The best performance in this group
**Chord**	Accuracy over 0.75
**Jaccard**	Accuracy over 0.75
**Dice**	Accuracy over 0.75
**Squared Chord DMs**	
**Squared Chord**	Accuracy over 0.75
Hellinger	Very similar to Squared chord distance

Continued on next page

Table S1: List of observed distance measures for the selection of reliable distance measures together with the explanation of chosen and discarded. Those marked in bold are included in this study. (Continued)

**Distance Measure**	**Explanation**
Matusita	Very similar to Squared chord distance
Bhattacharyya	Low performance
**Vicissitude DMs**	
**Vicis symmetric 1**	The best performance in the group
Vicis symmetric 2	Low performance
Vicis symmetric 3	Low performance
Vicis-Wave Hedges	Low performance
Max symmetric X^2	Low performance
Min symmetric X^2	Low performance
**Squared L_2 DMs**	
**Clark**	Accuracy over 0.75
**Divergence**	Accuracy over 0.75
**Squared Euclidean**	Accuracy over 0.75
**Average Euclidean**	Accuracy over 0.75
**Mean Censored Euclidean**	Accuracy over 0.75
Pearson X^2	Low performance
Neyman X^2	Low performance
Squared X^2	Low performance
Squared Chi-Squared	Low performance
Probabilistic Symmetric X^2	Low performance
Additive Symmetric X^2	Low performance
**Shannon Entropy DMs**	
**Jeffreys**	The best performance in the group
**Divergence**	Accuracy over 0.75
**Jensen difference**	Accuracy over 0.75
**Kullback-Leibler**	Used for optimization of t-SNE algorithm
Topsoe	Low performance
K divergence	Low performance
**Other DMs**	
**Average Distance (L_1, L_∞)**	Accuracy over 0.75
**WIAD**	Accuracy over 0.75
**Squared Pearson**	Accuracy over 0.75
**Correlation**	Accuracy over 0.75
**Pearson**	Accuracy over 0.75
**Motyka**	Accuracy over 0.75
**Hassanat**	Accuracy over 0.75
Kumar-Johnson	Low Performance
Taneja	Low Performance

Continued on next page

Table S1: List of observed distance measures for the selection of reliable distance measures together with the explanation of chosen and discarded. Those marked in bold are included in this study. (Continued)

**Distance Measure**	**Explanation**
Hamming	Low Performance
Meehl	Low performance
Hausdorff	Low performance
X^2 statistic	Low performance

2. SELECTION OF THRESHOLD VALUE FOR ELIMINATION

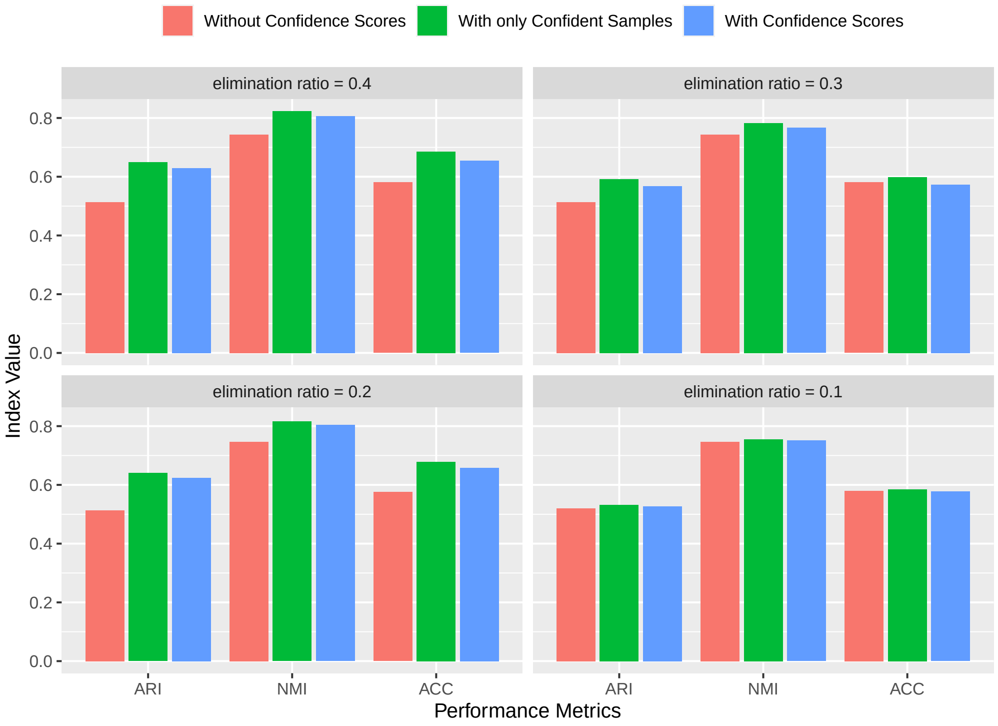

Fig. S1. Evaluation of the K-Means clustering performance on the whole dataset without confidence scores, on the dataset with only high-confident samples, and on the dataset where the eliminated samples are then positioned on the existing clusters, respectively. The evaluation metrics are calculated on the AMB18 dataset with the elimination ratios of 0.4, 0.3, 0.2, and 0.1

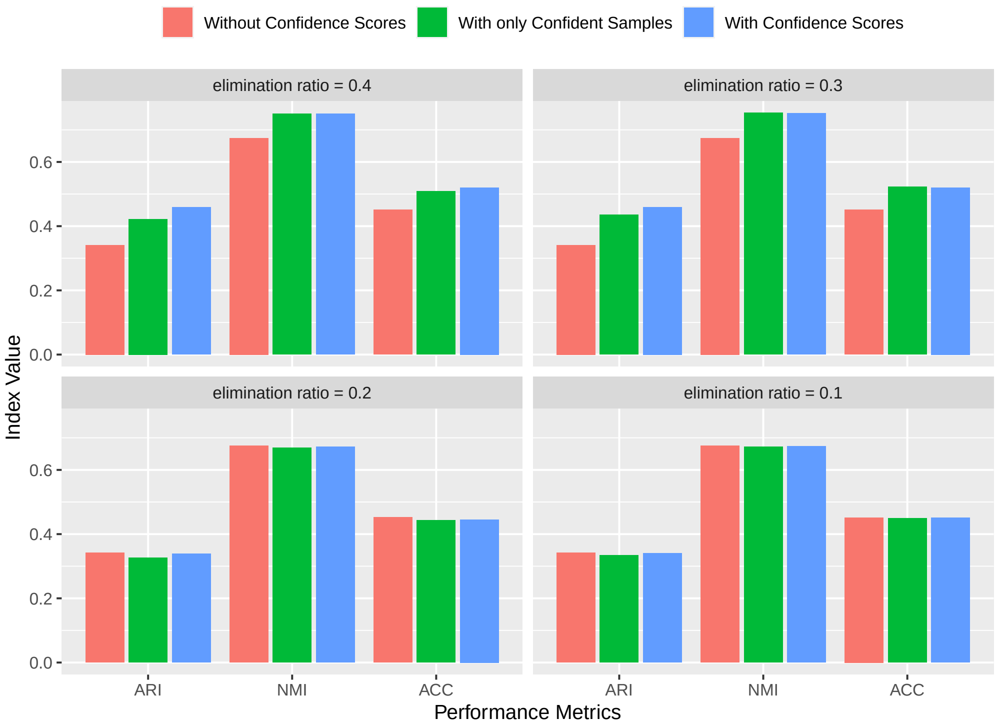

Fig. S2. Evaluation of the DBSCAN clustering performance on the whole dataset without confidence scores, on the dataset with only high-confident samples, and on the dataset where the eliminated samples are then positioned on the existing clusters, respectively. The evaluation metrics are calculated on the AMB18 dataset with the elimination ratios of 0.4, 0.3, 0.2, and 0.1
